# Swallowing characteristics in Down syndrome at an advanced age: a preclinical study in the Ts65Dn mouse model

**DOI:** 10.3389/fneur.2026.1703041

**Published:** 2026-01-21

**Authors:** Marziyeh Ostadi, Erin H. Fisher, Y. Eugene Yu, Nadine P. Connor, Tiffany J. Glass

**Affiliations:** 1 Department of Communication Sciences and Disorders, University of Wisconsin, Madison, WI, United States; 2 Department of Otolaryngology, Head and Neck Surgery, University of Wisconsin, Madison, WI, United States; 3 The Children’s Guild Foundation Down Syndrome Research Program, Department of Cancer Genetics and Genomics, Roswell Park Comprehensive Cancer Center, Buffalo, NY, United States

**Keywords:** aging, Down syndrome, mouse, swallowing, VFSS, Ts65Dn

## Abstract

Down syndrome (DS) occurs in approximately one in 800 births worldwide and is associated with various medical complications including swallowing impairments and dysphagia. Advances in childhood survival have led to an increased number of people with DS reaching older ages. Aging is generally accompanied by changes in sensory and motor functions, including those involved in swallowing. Swallowing impairments can lead to complications such as malnutrition, dehydration, and aspiration pneumonia. Despite the multifactorial nature of swallowing impairments in older people with DS, research in this area remains limited, with most studies focusing on pediatric populations. We hypothesized that aged Ts65Dn mice (mouse model of DS; 14 males, 15 females) would demonstrate significant impairments in swallowing performance on quantitative measures derived from videofluoroscopic swallow studies, relative to age-matched euploid controls (genetically typical mice; 14 males and 15 females). Statistical analyses included two-way ANOVA for genotype and sex effects. The Ts65Dn group exhibited significantly lower swallow rates, longer inter-swallow intervals (ISI), increased jaw cycle: swallow ratios (JSR), and lower jaw excursion rates (JER) than euploid controls. Body weight was significantly lower in the Ts65Dn group. These findings confirm persistent swallowing impairments in aged Ts65Dn mice, supporting their use as a model for studying swallowing mechanisms, provide critical insights into swallowing impairments in aging DS and support the need for specialized clinical interventions for swallowing disorders in this population.

## Introduction

Down syndrome (DS), resulting from trisomy of chromosome 21(HSA21) (1), is the most common genetic cause of intellectual disability (2). It occurs in approximately one in 800 births worldwide (3), and one in 582 births in recent years in the United States (4). DS is associated with various medical complications, including cardiovascular malformations, gastrointestinal atresias, neurodevelopmental disorders, feeding and swallowing disorders (3). Advances in childhood survival have shifted the age demographics of individuals with DS, leading to an increasing number of people reaching their 50s and 60s who can experience age-related health issues and atypical aging (5, 6).

Aging is generally accompanied by changes in sensory and motor functions, including those involved in swallowing (7). Swallowing impairments such as dysphagia affect 10 to 33% of older adults (8) and can lead to complications such as malnutrition, dehydration, and aspiration pneumonia (8). Age-related swallowing impairments may be further exacerbated by comorbid medical conditions in DS like Alzheimer’s disease (AD) (9). High prevalence of early-onset AD is commonly observed in adults with DS (6). AD affects over 90% of individuals with DS after age 40 largely due to the presence of the amyloid precursor protein (APP) gene located on the extra copy of chromosome 21 resulting from trisomy 21 (6, 9). As such, AD further exacerbates swallowing impairments (10).

Despite the multifactorial nature of swallowing impairments in people with DS at old ages, research in this area remains limited, with most studies focusing on pediatric populations (11–14). Furthermore, several barriers hinder research and clinical management of swallowing impairments in this population, including a lack of comprehensive and interdisciplinary perspectives (8), limited specialized care centers (15), and communication challenges between healthcare providers and individuals with DS, particularly those using modalities other than oral communication, such as augmentative and alternative communication (AAC) (16). Moreover, aging processes can cause limitations in physical function and reduced participation in community activities (17). Collectively, people with DS at older ages may face greater barriers to participating in research. Perhaps because of this, there is a lack of comprehensive data on swallowing-related outcomes in people with DS at advanced ages. This lack of information is limiting efforts to develop specific and effective management strategies.

To address these gaps, translational research using animal models offers a new avenue for investigating DS-related swallowing impairments (18, 19). The Ts65Dn mouse is widely used as a preclinical model for DS. The Ts65Dn mouse model recapitulates DS by carrying a partial trisomy that includes a segment from the distal end of mouse chromosome 16 and a centromeric region of chromosome 17 (20). This model exhibits several phenotypic characteristics that parallel those seen in humans with DS, including muscle weakness, reduced motor coordination, increased tongue muscle fatigue, mastication deficiency and interhemispheric connectivity defects (21–24). Our previous studies have also identified atypical swallowing performance in adult Ts65Dn mice (24, 25).

To investigate swallow function and physiology, the videofluoroscopic swallow study (VFSS) is a gold-standard diagnostic tool in clinical studies, assessing oropharyngeal and biomechanical function of swallowing (26). A murine VFSS protocol was previously developed to assess swallow parameters in mice (27) and has been used to study typically aging mice (28). Swallowing changes similar to presbyphagia in humans were identified in the aging mice, with delays and impairments observed across the oral, pharyngeal, and esophageal phases of swallowing (28). Specifically, this study reported significantly slower licking speed, longer pharyngeal and esophageal transit times, larger swallowed food boluses, and a higher rate of ineffective esophageal swallows in typically aging mice than younger mice (28). Although we know presbyphagia occurs in older typical mice, it is unknown how age impacts swallowing in DS mouse models. We hypothesized that aged Ts65Dn mice would demonstrate significant impairments in swallowing performance on quantitative measures derived from VFSS, relative to age-matched euploid controls (genetically typical mice).

## Materials and methods

### Mice

Ts65Dn mice were obtained from The Jackson Laboratory and bred by mating Ts65Dn females (stock #005252) with B6EiC3Sn. BLiAF1/J males (stock #003647) to produce the experimental mice used in this study. A total of 58 mice aged 20 months (ranging from 601 to 615 days, equivalent to advanced adult age in humans) were used in this study. The cohort was comprised of two genotypes—Ts65Dn (14 males, 15 females) and euploid controls (14 males and 15 females), all of which underwent videofluoroscopic swallow study (VFSS). All animal procedures were approved by the Institutional Animal Care and Use Committee (IACUC) of the University of Wisconsin School of Medicine and Public Health and conducted in accordance with the Guide for the Care and Use of Laboratory Animals (8th edition, The National Academies Press, 2011).

Mice were weaned on postnatal day 21 (P21) and maintained on a standard ad libitum hard pellet diet (Harlan Teklad 7,913). Mice also received water through a water bottle. All mice were housed in standard microisolator cages (7 × 5 × 11 inches) with corn cob bedding mixed with paper enrichment (SSP, Shepherd’s Cob +Plus). Environmental conditions were controlled with ambient temperature maintained between 68–79 °F and relative humidity between 30–70%. Mice were housed under a reverse 12-h light cycle with lights off from 9:00 a.m. to 9:00 p.m.

Mice were socially housed in same-sex mixed-genotype cages with up to 4 mice in a cage at a time, from weaning until their experimental endpoint. There were a few cases where males needed to be singly housed for a period of time prior to VFSS. These were primarily due to aggression toward cage mates, excessive barbering, or poor health. Once males were removed from a social cage they were not reintroduced. When applicable, females were re-housed with other females to avoid solitary housing in extenuating circumstances (i.e., attrition of cage mate), or if there was evidence of social stress such as excessive barbering.

### Videofluoroscopic swallow study (VFSS) acquisition

Swallowing function was assessed using VFSS. Animals were assigned auto-generated numerical identifiers at postnatal day 0 (P0), which were maintained throughout their lifespan to mask genotype information during imaging and data analysis.

Food for VFSS consisted of a 2:1 mixture of Fritos Mild Cheddar Cheese™ Dip and a Thin Honey 40% w/v barium sulfate oral suspension (Bracco Diagnostics, Varibar), resulting in a very thick puree consistency equivalent to IDDSI Level 4. Mice were acclimated to the cheese 3 days prior to imaging and were acclimated to barium mixed cheese 1 day prior to imaging. Mice were individually housed the night before imaging, and food was withheld overnight, with total food regulation not exceeding 24 h. Water remained available ad libitum.

VFSS was performed during voluntary feeding using a Genoray ZEN-7000 fluoroscopic x-ray system under red light. Lateral views were captured to include at minimum the entire head and, when feasible, the upper torso including the stomach. Imaging continued until several clear swallows were observed, with a minimum total recording time of 7 s. Recording was paused during any period when the mouse stopped eating, and imaging resumed upon recommencement of eating.

Imaging parameters included a 60 frames-per-second acquisition rate with a shutter speed of 16.66 milliseconds and a resolution of 768 × 896 pixels, using the PFV4 (Photon Fast Camera Viewer) software. X-ray settings included 47 kVp and 5.8 mA, with fluoroscopy mode engaged, a 4.5-inch collimator, and dynamic noise reduction set to high. Footage collection occurred between 6:00 a.m. and 12:00 p.m., with re-trials conducted for select animals between 3:00 p.m. and 4:00 p.m., as necessary. After imaging, mice were weighed prior to euthanasia.

### VFSS analysis

**Figure 1:**
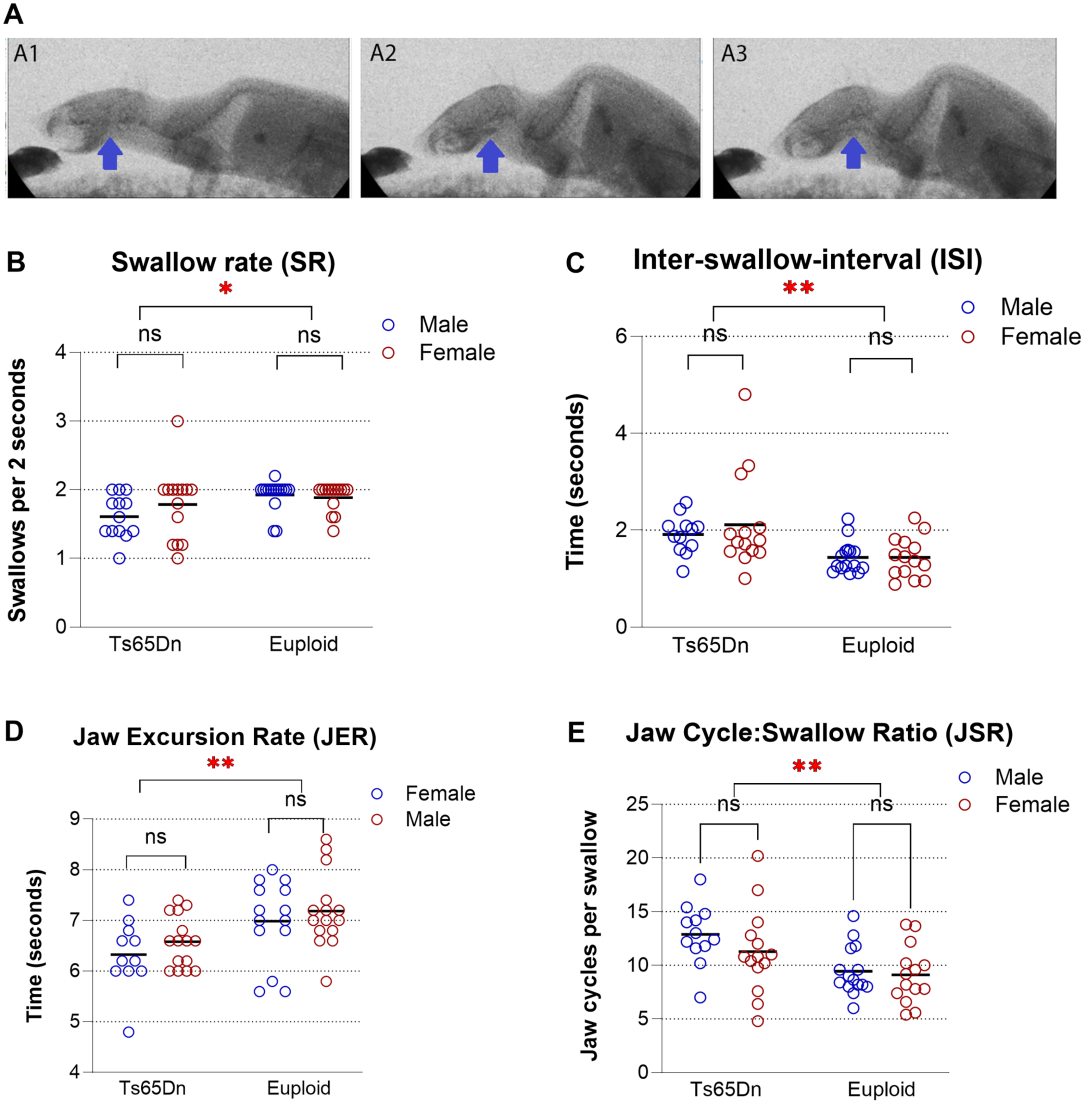
**(A)** Videofluoroscopic swallow study (VFSS). A 20-month-old male Ts65Dn mouse during feeding. **(A1)** Immediately prior to initiating the oral preparation of the bolus. **(A2)** A frame immediately prior to swallow initiation. **(A3)** A frame immediately following swallow initiation. **(B–E)** Videofluoroscopic swallow study analysis in 20-month-old Ts65Dn in comparison to euploid: **(B)** Swallow rate, the Ts65Dn group exhibited a significantly lower SR than the euploid group, independent of sex **(C)** Inter-swallow interval (ISI) values were significantly higher in the Ts65Dn group than the euploid group, regardless of sex **(D)** Jaw excursion rate (JER) was significantly lower in the Ts65Dn group than the euploid group **(E)** Jaw cycle: swallow ratio (JSR) the Ts65Dn group demonstrated significantly higher JSR values than the euploid group. Each symbol indicates a single mouse. Bars show the mean. *p ≤ 0.05, **p ≤ 0.01, ns = not significant.

Videofluoroscopic recordings (Figure 1A) were manually analyzed in ImageJ software according to previously validated protocols (27) to quantify swallowing function. Six parameters were extracted: first, swallow rate (SR), defined as the number of swallows per 2 s of uninterrupted feeding; second, inter-swallow interval (ISI), defined as the time between two consecutive swallows, was calculated individually by measuring the interval between each pair of consecutive swallows; Third, jaw excursion rate (JER), the number of jaw opening–closing cycles per second; fourth, jaw cycle: swallow ratio (JSR), calculated as the number of jaw excursions per ISI. Each of the four parameters was measured for five instances per animal and averaged to produce a final data point for statistical analysis. Delta ISI (ΔISI) was defined as the difference between the final swallow ISI and initial swallow ISI within an imaging session; and Delta JSR (ΔJSR) was defined as the difference between the final swallow JSR and initial swallow JSR within an imaging session.

In the context of thick puree feeding, the JSR served a role similar to the lick–swallow ratio used in liquid-based assessments (27). JSR was used here to accommodate the possibility of additional oral motor behaviors such as jaw cycles due to oral processing other than, or in addition to, licking (25).

### Statistical analysis

Inter-rater reliability was assessed using the Intraclass Correlation Coefficient (ICC). Rater 1 independently evaluated all VFSS recordings. Rater 2 analyzed a randomly selected subset of 18 videos (>20% of the total samples). To evaluate the normality of data, Shapiro–Wilk test was used. Measures that adhered to the normality assumption were analyzed using parametric ANOVA, whereas measures that violated the normality assumption were analyzed using a ranked ANOVA approach. To evaluate the effects of genotype and sex on swallowing function, statistical analysis was performed using two-way analysis of variance (two-way ANOVA) for weight, SR, ISI, JER, and JSR, ΔISI and ΔJSR. To evaluate the impact of aging (5 months vs. 20 months) and genotype on swallow function, two-way ANOVA was used for ISI measures in genotype groups with pooled sexes. All data analyses were conducted using SAS version 9.4 (SAS Institute, Inc., Cary, NC) and GraphPad Prism (GraphPad Software Inc., San Diego, CA, USA). An alpha level of 0.05 was used to define statistical significance.

## Results

In the study, 53 of 58 mice were included in the analysis: Ts65Dn (12 males, 14 females) and euploid controls (13 males and 14 females). Five mice were excluded which represented both genotypes and sexes (1 Male Euploid, 1 Female Euploid, 2 Male Ts65Dn and 1 Female Ts65Dn). They were excluded because they exhibited fewer than five swallows per meal during VFSS imaging, typically due to being too weak, non-compliant, or excessively mobile to complete a sufficient number of swallows.

### Swallow functions

The analysis demonstrated inter-rater agreement, as measured by ICC, ranging from 0.85–0.98 agreement across all measured swallowing variables, indicating good to excellent reliability.

Swallow Rate (SR): The Ts65Dn group exhibited a significantly lower SR than the euploid group, independent of sex [*F* (1, 49) = 6.52, *p* = 0.0138; Figure 1B]. No significant differences in SR were observed between male and female groups within either genotype, and the interaction between genotype and sex was not significant, suggesting a consistent genotype effect across sexes.

Inter-Swallow Interval (ISI): ISI values were significantly higher in the Ts65Dn group than the euploid group, regardless of sex [*F* (1, 49) = 11.14, *p* = 0.0016; Figure 1C]. No significant differences were found due to sex, and the genotype-by-sex interaction was not significant, indicating a stable genotype effect.

Jaw Excursion Rate (JER): JER was significantly lower in the Ts65Dn group than the euploid group [*F* (1, 49) = 9.90, *p* = 0.0028; Figure 1D]. Sex differences did not reach statistical significance. The genotype-by-sex interaction was also not significant.

Jaw cycle: swallow ratio (JSR): The Ts65Dn group demonstrated significantly higher JSR values than the euploid group [*F* (1, 49) = 10.32*, p* = 0.0023; Figure 1E]. Sex did not significantly influence JSR, and no significant interaction between genotype and sex was detected.

**Figure 2:**
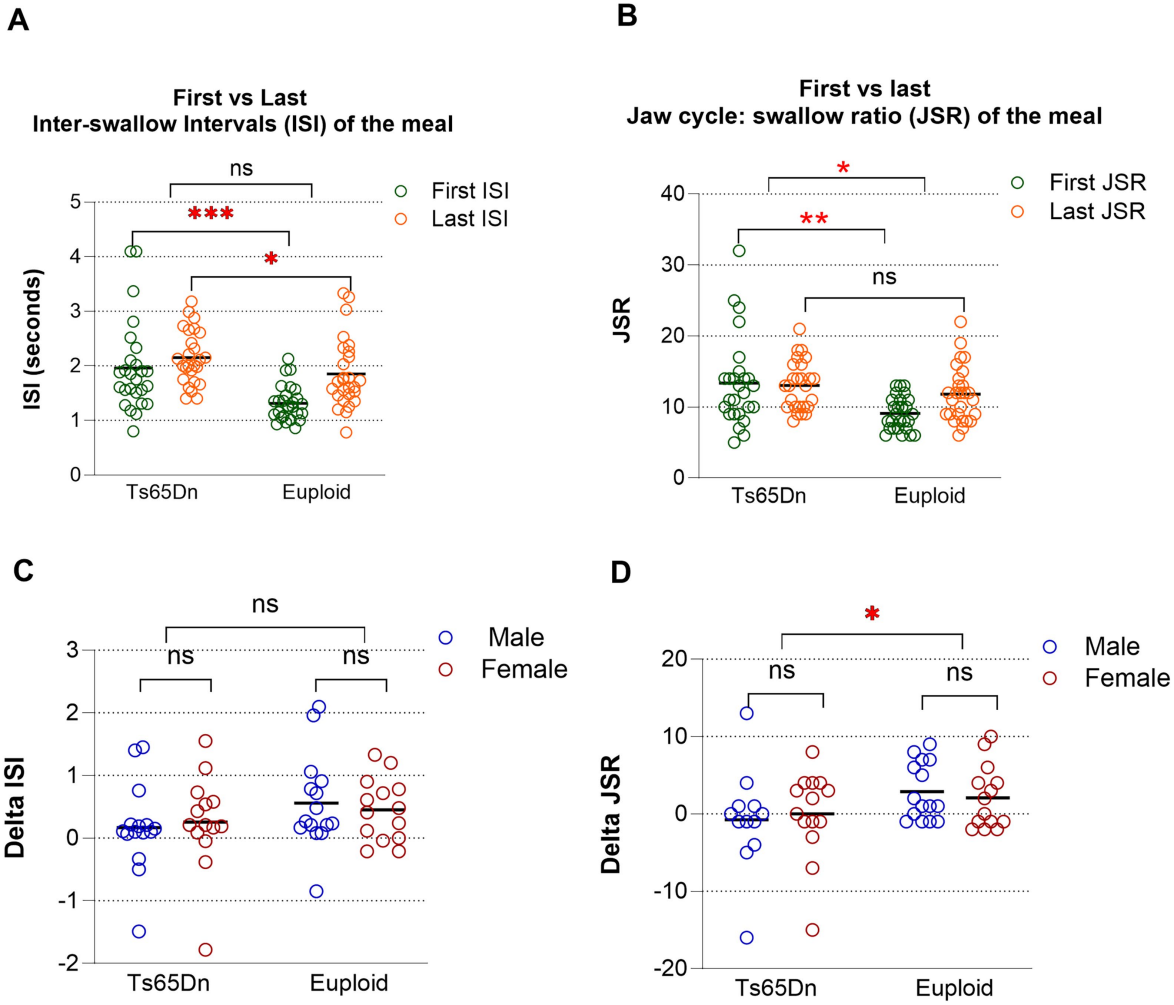
Videofluoroscopic swallow study analysis in 20-month-old Ts65Dn in comparison to euploid: **(A)** First inter-swallow interval (ISI) versus last ISI, data pooled across sexes. **(B)** First jaw cycle: swallow ratio (JSR) versus last JSR, data pooled across sexes. **(C)** Delta ISI (change in ISI between the first and last swallows of a meal) in Ts65Dn and euploid mice. **(D)** Delta JSR (change in JSR between the first and last swallows of the meal). Each symbol indicates a single mouse. Bars show the mean. *p ≤ 0.05, **p ≤ 0.01, ***p ≤ 0.001, ns = not significant.

Change in ISI Over Time (ΔISI): Swallow function may change during the course of a meal due to factors such as satiation or fatigue; this can be particularly true for humans at advanced age (29). Therefore, the assessments were performed at both the onset and the end of each VFSS session to obtain an indication of change in function over the course of a meal. Ts65Dn showed significantly higher First ISI [*F*(1, 49) = 16.31, *p* = 0.0002; Figure 2A**]** and higher last ISI [*F*(1, 49) = 4.70, *p =* 0.0350; Figure 2A] than Euploid control. While genotypes appeared to influence ΔISI, with the Ts65Dn group showing a smaller increase, than the euploid group, this effect did not reach statistical significance [*F* (1, 49) = 3.48, *p* = 0.0683] (Figures 2A,C). Sex had no significant effect, and the interaction term was also non-significant. No significant pairwise group differences were identified. The model accounted for only 7.6% of the variance (R^2^ = 0.076), suggesting that additional factors may contribute to ISI variability.

Change in JSR Over Time (ΔJSR): Similar to ISI, JSR was also significantly affected in both genotypes, with Ts65Dn showing a higher First JSR than the Euploid control [*F* (1, 49) = 11.65, *p* = 0.0013; Figure 2B]. Last JSR was slightly higher in Ts65Dn than Euploid control, but did not reach statistical significance (Figure 2B). Genotype had a statistically significant effect on the change in JSR over time [*F* (1, 49) = 4.86, *p* = 0.0323], with the Ts65Dn group exhibiting a smaller increase than the euploid group (Figures 2B,D). Sex did not significantly affect ΔJSR, and the genotype-by-sex interaction was not significant. The model explained 9.7% of the variance in ΔJSR, indicating that other unmeasured variables may influence this outcome.

### Body weight

**Figure 3:**
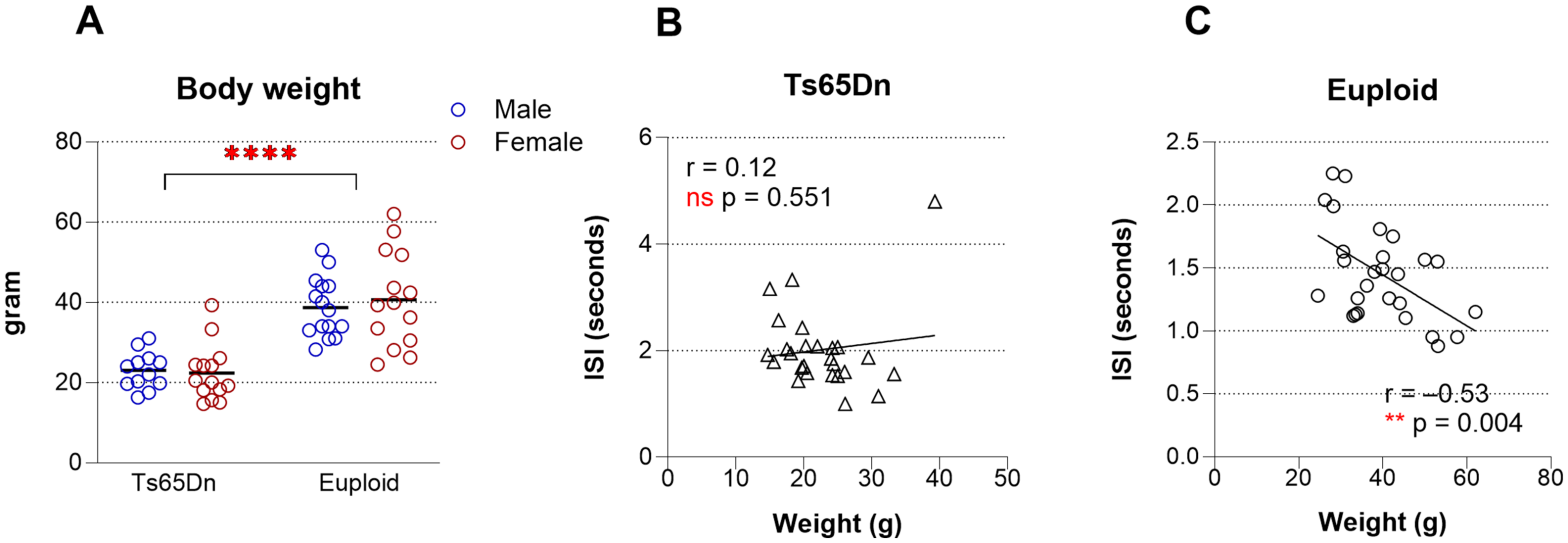
Body weight analysis in 20-month-old mice. **(A)** Comparison of body weight between aged euploid and Ts65Dn mice, indicating that euploid mice weighed substantially more than their Ts65Dn counterparts. **(B)** Correlation between body weight and inter-swallow interval (ISI) in Ts65Dn mice, suggesting no meaningful linear association between body weight and ISI in this group. **(C)** Correlation between body weight and ISI in euploid mice, indicating that higher body weight was associated with reduced ISI. Each symbol indicates a single mouse. Bars show the mean. ****p ≤ 0.0001.

During aging, unintentional weight loss is associated with swallowing difficulties (30). In this study, body weights in the 20-month-old euploid and Ts65Dn group were assessed to determine age-related genotype differences and explore associations between body weight and ISI, as a measure of swallowing function. ISI was chosen as representative of the various parameters measured in this study because it was the most robust measure for the detection of genotype-specific differences, with the lowest *p*-value and the greatest *F* value of the VFSS measures. As shown in Figure 3A, the euploid group exhibited a significantly higher mean body weight than the Ts65Dn group. This difference was statistically significant [*F* (1,49) = 40.66, *p <* 0.0001], indicating a pronounced reduction in body mass associated with the Ts65Dn genotype. In contrast, there was no significant main effect of sex in body weight.

To assess whether body weight was associated with ISI, Pearson correlation analyses were performed separately for each genotype. In the Ts65Dn group (n = 26), no significant relationship was found between body weight and ISI, suggesting the absence of a meaningful linear association (Figure 3B). In contrast, the euploid group (n = 27) demonstrated a moderate and statistically significant negative correlation between body weight and ISI with 28.6% of ISI variance explained by body weight (Figure 3C).

### Impact of aging on swallow function

**Figure 4:**
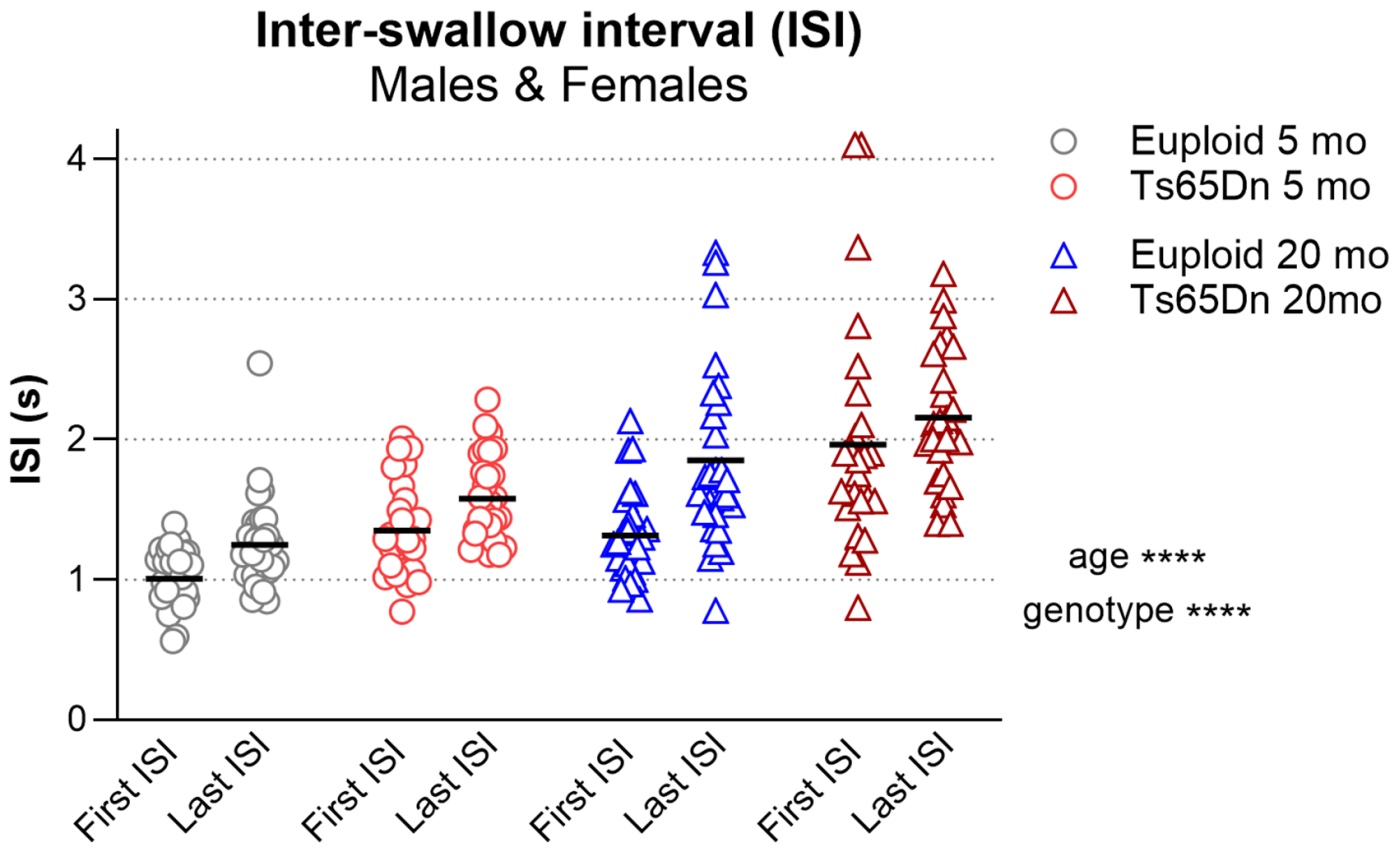
First vs last ISI comparison between 5-month (adult) and 20-month (old adult) mice. The figure illustrates a comparison between the first and last ISI recorded during mealtime in VFSS recordings. ISI data from 5-month-old mice, reported in our previous study (24) are compared to those from 20-month-old mice examined in the current study (*p <* 0.0001). Sexes have been pooled. Each symbol indicates a single mouse. Bars show the mean. ****p ≤ 0.0001.

To consider the impact of the aging process on swallow function in Ts65Dn, current data from 20-month-old mice were analyzed in comparison to historical data from 5-month-old mice in a previously reported study (24) (Figure 4), using nearly identical VFSS analysis paradigms. As ISI is a robust measure reflecting factors that can impact both SR and JSR, ISI was selected for expanded analysis of the aging effect on swallow function. Overall, ISI was significantly impacted by both age and genotype in the absence of significant interactions between age and genotype. The aging process results in significantly higher values for the general ISI measure [*F*(1, 105) = 54.34, *p <* 0.0001], as well as for both the first ISI of the meal [*F*(1, 105) = 32.71, *p <* 0.0001] and the last ISI of the meal [*F*(1, 105) = 51.88, *p <* 0.0001]. In addition, the Ts65Dn genotype results in significantly higher values for the general ISI measure [*F*(1, 105) = 31.69, *p <* 0.0001], as well as for both the first ISI of the meal [*F*(1, 105) = 39.18, *p <* 0.0001] and the last ISI of the meal [*F*(1, 105) = 20.73, *p <* 0.0001]. Collectively, the adult aging process and the Ts65Dn genotype both independently impact swallow function by increasing the time interval between consecutive swallows during continuous eating. This results in compounded impacts of both advanced age and genotype in aged Ts65Dn, such that aged Ts65Dn have the highest ISI values of all age/genotype combinations evaluated.

## Discussion

This study investigated the swallowing phenotype in aged Ts65Dn mice, a model of DS, in comparison to typically aging euploid mice. We hypothesized that aged Ts65Dn mice would demonstrate significant impairments in swallowing performance on quantitative measures derived from VFSS, relative to age-matched euploid controls. Specifically, we hypothesized that aged Ts65Dn mice would exhibit significantly reduced swallow rate, increased inter-swallow interval, decreased jaw cycle: swallow ratio, and prolonged jaw excursion rate, indicative of delays or dysfunction in the oral and pharyngeal phases of swallowing during feeding. The findings allowed us to accept our hypothesis for five out of the six parameters, providing preliminary evidence supporting the validity of aged Ts65Dn mice as a translational murine model for investigating swallowing disorders in aging DS. This study demonstrated that aged Ts65Dn mice exhibited significantly lower swallowing rates (SR) and longer inter-swallow intervals (ISI) than euploid controls, indicating impaired swallowing function in these mice characterized by longer durations before each swallow.

The observed impairment in swallowing function in our study of Ts65Dn is not only congruent with prior murine studies (24, 25, 28), but also with human research on aging individuals with DS (9). Healthy aging is characterized by a gradual decline in skeletal muscle mass, strength, and efficiency (31), which encompasses masticatory, facial and tongue muscles (32, 33). In addition, there is an increase in connective tissue within the muscles involved in the swallowing mechanism that can contribute to stiffness, reduced mobility, and diminished flexibility (9), thereby slowing the passage of the bolus through the oropharynx (9). There is also reason to believe that the aging process may be more pronounced and atypical in DS (34).

Our study showed that the Ts65Dn genotype has significantly higher jaw cycle: swallow ratio (JSR) values than the euploid genotype. The result indicates that older Ts65Dn mice have more jaw cycles before each swallow, consistent with longer oral processing times of the bolus before swallowing (25). Similarly, in a prior study of the oral stage of swallowing in typical older mice, lick rate, defined as the frequency of tongue and jaw movements per second, was significantly slower in typical aged mice than their younger counterparts (28). In rodents, licking is a critical oromotor behavior for nutrient acquisition, whereas in humans, liquid intake is typically achieved through cup or straw drinking (35). Although these behaviors are species-specific and differ in their observable execution, both involve complex, coordinated movements of the tongue, jaw, and other oral structures to fulfill the shared functional objective of nutritional intake. The higher jaw cycle per swallow ratio also aligns with a human study reported that chewing disturbances in adults with DS are related to disorganized masticatory muscle activity and tongue thrusting (36). Our finding of higher jaw cycle per swallow rate in older Ts65Dn mice is also consistent with a report in healthy older humans showing that while aging has minimal effect on the ability to adapt to changes in food hardness, older individuals tend to perform more chewing cycles with longer durations per cycle (37). It seems older adults with DS also adapt their feeding behavior. There are limited studies on swallowing function in aging individuals with DS, however, a review study reported that as children with DS develop, they undergo physiological and anatomical changes to eating and swallowing, that may require them to adopt compensatory strategies such as tongue protrusion and tongue thrusting to compensate for efficient mastication and control of the bolus (6, 9). As such, the 20-month mice in the study showed more jaw cycles per swallow perhaps to compensate for their swallow dysfunction.

Comparison of results from this study on 20-month old Ts65Dn to results of previous studies of 5-month old Ts65Dn (24) suggests that aging may impact swallow function in DS in at least two different ways. First, there may be aging-related swallow phenotypes that are unique to DS, which are not present in age-matched controls. This is suggested by the fact that in previous swallowing studies on 5-month-old mice (24, 25) there was no significant difference in jaw excursion rate (JER) between Ts65Dn and control. However, a significant difference in JER between Ts65Dn and control was found at 20 months. This suggests that significantly lower JER is an age-related swallowing behavior unique to the 20-month Ts65Dn mice. In contrast, ISI showed significant differences between Ts65Dn and euploid not only at 5-months but also at 20-months in our analysis. It showed impairment present in Ts65Dn at adulthood and worsening with aging (24). In our analysis, we found that Ts65Dn mice exhibited significantly longer ISI than age-matched euploid controls at both ages (24). Moreover, aged groups, both those with and without DS, demonstrated significantly prolonged ISI compared to their respective younger counterparts (24). This suggests the aging process may exacerbate swallowing phenotypes that are already present in DS at younger adult ages.

Individuals with DS may experience biologically accelerated aging, however chronological age is typically used as a reference for comparison. In this context, 5-month-old mice correspond roughly to 30-year-old humans, and 20-month-old mice correspond to 70–80-year-old humans (38). It is important to note that chronological age does not fully capture the biological aging observed in individuals with DS. Precocious biological aging may happen in some body systems of people with DS such as muscles, movement, metabolism, kidneys, heart, hearing and vision (39). They may show earlier age-related decline, and conditions like Alzheimer’s disease may appear at younger chronological ages. In our study, we used chronological age to establish a consistent aging scale while acknowledging that some systems in Ts65Dn mice may exhibit accelerated biological aging.

The findings of this study reveal a robust genotypic effect on body weight in aged mice, with Ts65Dn mice exhibiting significantly lower weights than their euploid counterparts. ISI showed no correlation with body weight in Ts65Dn mice and the link between weight and ISI appeared unclear. In contrast, a significant inverse relationship between weight and ISI was observed in typical euploid mice. This relationship showed the heavier euploid mice tended to exhibit shorter ISI, suggesting increased swallowing efficiency correlated with greater body mass. This suggests the possibility that in mice, as in humans, reduced swallowing efficiency can be implicated in reduced body weights. However, the implication of these correlation findings for Ts65Dn should be interpreted with caution due to the apparent range in Ts65Dn body weight data, which may impact correlation values and make it difficult to conclude with certainty that reduced swallowing efficiency is a cause of reduced body weights in Ts65Dn. Previous reports of altered swallow function in adult mouse models of DS also showed significant weight differences between 5-month-old Ts65Dn mice and the euploid controls (25). This is additionally reflected in a human study that identified unintentional weight loss as an early clinical feature of Alzheimer’s disease in individuals with DS (40). A previous study found that adults with DS may experience weight loss earlier in life than adults with other forms of intellectual disability, despite exhibiting greater levels of obesity in early adulthood (41).

Despite its strengths, this study has some limitations. The Ts65Dn mouse model carries a partial trisomy that includes a segment of mouse chromosome 16, syntenic to human chromosome 21, and a segment of chromosome 17, which is not syntenic to human chromosome 21 (42). This genetic composition complicates the interpretation of phenotypic outcomes, including those observed in VFSS. The study did not determine which specific region of the partial trisomy underlies the VFSS findings in Ts65Dn mice. Notably, the inclusion of chromosome 17 may influence certain phenotypes (42), though its specific impact on swallowing function remains unclear.

Future studies using the Ts65Dn; Df(17)2 model, which corrects the triplicated segment of chromosome 17, could help clarify the genetic basis of the observed swallowing abnormalities. Ts66Yah could also be used for this purpose. The key difference between Ts65Dn; Df(17)2 and Ts66Yah lies in their normalization strategies: Ts65Dn; Df(17)2 achieves normalization through a *trans* deletion on the homologous chromosome, whereas Ts66Yah uses a *cis* deletion directly on the Ts(17(16))65Dn chromosome (42, 43). Additionally, a previous study highlighted that some genes syntenic to mouse chromosome 17 may contribute to developmental feeding difficulties (44). This underscores the importance of studying the role of mouse chromosome 17 in VFSS outcomes, which could have broader implications for understanding feeding disorders of other developmental disorders beyond DS.

## Conclusion

This study confirms that older Ts65Dn mice exhibit significant impairments in swallowing function compared to euploid controls. These impairments are evidenced by reduced swallowing rates, longer inter-swallow intervals, increased oral processing time by increased jaw cycles per swallow, and lower jaw open-close rates or jaw excursion rates. These findings are broadly consistent with previous observations in younger adult Ts65Dn mice and align with reported swallowing and chewing difficulties in individuals with DS. The elevated jaw cycles per swallow suggest compensatory behaviors to manage bolus formation and transport, likely due to oromotor inefficiencies. Furthermore, the reduced increase in jaw cycles over the course of a meal (Delta JSR) in Ts65Dn mice may reflect limitations in their masticatory system that are unaffected by meal duration. The absence of sex-based differences across all measured variables suggests that these swallowing impairments are primarily genotype-related rather than sex-dependent. Altogether, this study highlights persistent swallow impairments in aging DS, offering valuable insights into the chronological, functional, and biological factors contributing to feeding and swallowing disorders in older adults with DS. These findings also inform clinical approaches to provide specialized intervention for swallowing disorders in this population.

## Data Availability

The datasets presented in this study can be found in online repositories. The names of the repository/repositories and accession number(s) can be found at: Glass TJ, Connor NP, Fisher EH, Marziyeh O. Swallow function in the adult Ts65Dn mouse model of Down Syndrome. MPD: Glass1. Mouse Phenome Database web resource (RRID: SCR_003212), The Jackson Laboratory, Bar Harbor, Maine USA (https://phenome.jax.org/projects/Glass1).
